# 21-Gene Recurrence Score Assay Could Not Predict Benefit of Post-mastectomy Radiotherapy in T1-2 N1mic ER-Positive HER2-Negative Breast Cancer

**DOI:** 10.3389/fonc.2019.00270

**Published:** 2019-04-16

**Authors:** Wen-Wen Zhang, Qin Tong, Jia-Yuan Sun, Xin Hua, Zhi-Qing Long, Jia-Peng Deng, Yong Dong, Feng-Yan Li, Zhen-Yu He, San-Gang Wu, Huan-Xin Lin

**Affiliations:** ^1^State Key Laboratory of Oncology in Southern China, Department of Radiation Oncology, Collaborative Innovation Center for Cancer Medicine, Sun Yat-sen University Cancer Center, Guangzhou, China; ^2^Department of Radiation Oncology, The First Affiliated Hospital of University of South China, Hengyang, China; ^3^Department of Oncology, Dongguan Third People's Hospital, Affiliated Dongguan Shilong People's Hospital of Southern Medical University, Guangdong, China; ^4^Department of Radiation Oncology, Xiamen Cancer Hospital, The First Affiliated Hospital of Xiamen University, Xiamen, China

**Keywords:** breast cancer, micrometastasis of lymph nodes, post-mastectomy radiotherapy (PMRT), 21-gene recurrence score (RS) assay, surveillance, epidemiology, and end results (SEER) database

## Abstract

**Introduction:** It is still controversial whether post-mastectomy radiotherapy (PMRT) is necessary for women with T1-2 N1mic ER-positive HER2-negative breast cancer. The 21-gene recurrence score (RS) assay has been validated in T1-2 N1 breast cancer to be prognostic of locoregional recurrence (LRR) and overall survival (OS). This study aims to evaluate the predict value of 21-gene recurrence score assay for the benefit of PMRT in T1-2 N1mic ER-positive HER2-negative breast cancer.

**Methods:** A population-based cohort study was performed on women with T1-2 N1mic ER-positive HER2-negative breast cancer who underwent mastectomy and were evaluated using the 21-gene RS in the Surveillance, Epidemiology, and End Results (SEER) registry between 2004 and 2015. Clinical characteristics as well as OS and breast cancer-specific survival (BCSS) were compared between patients with and without PMRT in patients with a Low-, Intermediate-, and High-RS. Multivariate COX regression analysis was performed to investigate if the 21-gene RS assay could predict benefit of PMRT in this group of breast cancer patients.

**Results:** A total of 1571 patients met the criteria of our study and were enrolled, including 970 patients in the Low-Risk group (score <18), 508 in the Intermediate-Risk group (score 18–30), and 93 patients in the High-Risk group (score >30). In the High-Risk group, there were more patients with age ≥50 (87.0 vs. 64.3%, *P* = 0.040) and received chemotherapy with a borderline significance (91.3 vs. 72.9%, *P* = 0.066) in the PMRT subgroup than in the no PMRT subgroup. In all three groups, OS was comparable between the PMRT subgroup and the no PMRT subgroup. Furthermore, multivariate analysis did not show any OS benefit for PMRT based on the 21-gene recurrence score.

**Conclusion:** This study showed that the 21-gene RS assay was not able to predict the benefit of PMRT for OS in women with T1-2 N1mic ER-positive HER2-negative breast cancer. However, further prospective larger sample-size trials are warranted to determine if a benefit exists.

## Introduction

According to the latest Global Cancer Statistics 2018, breast cancer is still the most frequent cancer and the leading cause of cancer death among females worldwide (1). It is estimated that there will be about 2.1 million newly diagnosed female breast cancer cases and 627,000 cancer death in 2018 (1). The use of screening tests for breast cancers provides better opportunities for patients to detect disease at an earlier stage and obtain more effective treatment with fewer side effects. As reported by the National Cancer Institute (NCI) in the Surveillance, Epidemiology, and End Results Program (SEER), about 93% of female breast cancers are diagnosed at localized or regional stage^1^. For early-stage breast cancer patients, three landmark clinical trials have demonstrated that post-mastectomy radiotherapy (PMRT) reduces locoregional recurrences (LRRs) and improves overall survival (OS) in high-risk patients, namely those with pathologically involved axillary nodes or with large tumor size (>5 cm) (2–4). And subsequently, subgroup analysis of DBCG 82 b & c trials (5) and meta-analyses from European Organization for Research on Treatment of Cancer (EORTC) (6) also showed the same positive effect of PMRT in patients with 1-3 positive nodes, even when systemic therapy was given. However, there is still controversy regarding the universal application of PMRT in all patients with T1-2 and 1-3 positive nodes. A joint statement by ASCO, ASTRO, and SSO proposes that some subsets of these patients are likely to have very low risk of LRR and the absolute benefit of PMRT might be outweighed by its potential toxicities (7). For instance, one group consists of patients with ER positive, HER2 negative, T1-2N1mic disease. The prognosis of patients with only micrometastasis of lymph nodes might lie between those with N0 disease and those with lymph node macrometastases, as studies showed inconsistent results (8–16). The 21-gene Recurrence ScoreTM (RS) assay (Genomic Health, Redwood City, CA) has been validated in T1-2 N1 breast cancer to be prognostic of LRR, disease-free survival (DFS), and OS (17–21). Recently, a study on the benefit of PMRT in T1-2 N1 breast cancer based on the 21-gene RS assay showed that patients with a low RS might derive the greatest survival benefit from PMRT while those with an intermediate or high RS did not (22).

To further investigate if the 21-gene RS may help in making the decision of whether or not to use PMRT in patients with ER positive, HER2 negative, T1-2N1mic disease, we conducted a population-based study based on data from the SEER database.

## Materials and Methods

### Study Population

Women with pathologic ER positive, HER2 negative, T1-2N1mic disease who underwent mastectomy and were evaluated using the 21-gene RS in the SEER registry between 2004 and 2015 were included. TNM stage of all patients was reassessed according to the 8th edition of the American Joint Commission on Cancer (AJCC) staging system. Inclusion criteria were as follows: (1) female gender; (2) stage T1-2 N1mic M0 disease based on the 8th edition of AJCC staging system; (3) ER-positive and HER2-negative disease; (4) underwent mastectomy; and (5) known radiation status. Exclusion criteria included: (1) radiotherapy performed before or during surgery; and (2) follow-up time <2 months.

We obtained access to the de-identified linked dataset after SEER approval of a custom data request and signature of a Data-Use Agreement. For analyses of de-identified data from the SEER registry, local institutional review board approval and informed consent were not required. Clinical information was obtained from the database, including patients' characteristics (e.g., gender, age at diagnosis, race), tumor characteristics (e.g., the Tumor, Node, and Metastasis (TNM) stage, histologic tumor type, histologic grade, ER status, PR status, human epidermal growth factor receptor 2 (HER2) status, the 21-gene RS), treatments (type of surgery, use of chemotherapy, use of radiotherapy), year of diagnosis, vital status, and survival months. The primary predictor variables of interest in this study were the 21-gene RS and PMRT. RS was defined as “Low-Risk” (score <18), “Intermediate-Risk” (18-30), or “High-Risk” (>30).

The primary endpoint was (OS) and the second endpoint was breast cancer-specific survival (BCSS). OS was defined as time from initial diagnosis to the date of death of any cause or last follow-up. BCSS was defined as time from initial diagnosis to the date of death of breast cancer, or death of any other cause or the last follow-up.

### Statistical Methods

We employed the SPSS version 23 statistical software (IBM Corporation, Armonk, NY, USA) to perform statistical analysis. Baseline clinical characteristics were compared using the independent sample *t*-test or Mann-Whitney *U*-test for continuous variables and the χ2 test or Fisher's exact test for categorical variables when appropriate. Survival analysis was performed using the Kaplan-Meier method and compared using the log-rank test for univariate analysis. Multivariate analysis was performed using the forward stepwise Cox proportional hazards method. A two-sided *P* < 0.05 was considered significant. Estimates were reported with the 95% confidence interval (CI) where appropriate.

## Results

**Figure 1:**
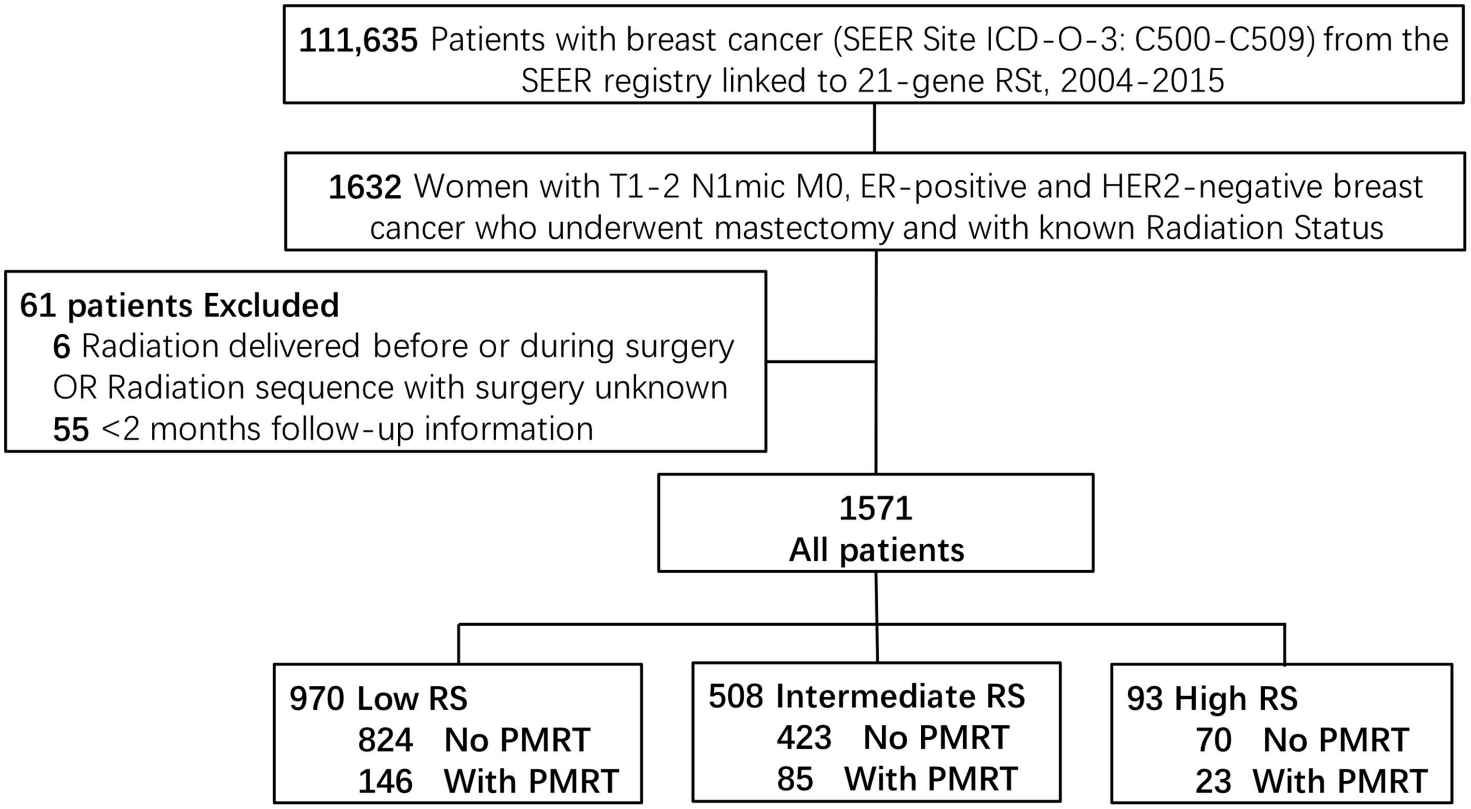
Diagram of cohort selection within the SEER database. TNM stage was according to the 8th edition of the American Joint Commission on Cancer (AJCC) staging system. T1-2, Tumor stage 1 or 2; N1mi, Nodal stage micrometastatic disease; M, Metastasis stage; ER, Estrogen Receptor; HER2, human epidermal growth factor receptor type 2; RS, 21-gene Recurrence Score Assay risk group; PMRT, Post-mastectomy radiotherapy.

A total of 111,635 patients were diagnosed with breast cancer and also with a documented 21-gene RS during 2004 to 2015 in the SEER database. Among those patients, 1632 women had T1-2 N1mic M0, ER-positive and HER2-negative disease and received mastectomy with the status of radiotherapy known. After exclusion of six patients whose radiation was delivered before or during surgery or with unknown sequence with surgery, and 55 patients whose follow-up time was <2 months, a total of 1,571 patients met the criteria of our study and were included. There were 970 patients in the Low-Risk group (score <18), 508 in the Intermediate-Risk group (score 18–30), and 93 patients in the High-Risk group (score >30). One hundred and forty-six (15.1%) patients in the Low-Risk group, 85 (16.7%) in the Intermediate-Risk group, and 23 (24.7%) in the High-Risk group received PMRT (*P* = 0.049) (Figure 1).

### Baseline Clinical Characteristics

Baseline clinical characteristics of the Low-, Intermediate-, and High-Risk group are listed in Table 1. In all three groups, the median ages of patients between the PMRT subgroup and the No PMRT subgroup were all comparable (*P* = 0.873, 0.322, 0.208). However, in the High-Risk group, there were more patients with age ≥50 in the PMRT subgroup than in the No PMRT subgroup (87.0 vs. 64.3%, *P* = 0.040). In the Low-Risk group, more patients in the PMRT subgroup had T2 disease (52.1 vs. 34.2%, *P* = 0.000). Similarly, more patients in the PMRT subgroup in the Low-Risk group had Grade 3 disease. While in the Intermediate-Risk and High-Risk group, there was no significant difference in terms of T stage or histologic grade between the PMRT subgroup and No PMRT subgroup. In addition, more patients in the PMRT subgroup were diagnosed in 2014 or after in the Intermediate-Risk group (41.2 vs. 28.8%, *P* = 0.025). Other variables, including race and histology type, were all comparable between the PMRT subgroup and No PMRT subgroup in all three groups.

**Table 1:** Baseline clinicopathological characteristics and treatment information of the PMRT subgroup and the No PMRT subgroup in the Low-, Intermediate-, and High-Risk group.

**Variable**	**Low-Risk**			**Intermediate-Risk**			**High-Risk**		
	**No PMRT (*N* = 824)**	**PMRT (*N* = 146)**	***P***	**No PMRT (*N* = 423)**	**PMRT (*N* = 85)**	***P***	**No PMRT (*N* = 70)**	**PMRT (*N* = 23)**	***P***
**Follow-up (months)**	30 (2–71)	28 (2–71)	0.323	32 (2–71)	28 (2–71)	0.235	31.5 (2–71)	26 (4–64)	0.081
**Age (Year)**									
Median (Range)	57 (24–85)	57 (33–82)	0.873	56 (20–86)	55 (30–88)	0.322	54.5 (23–86)	59 (37–85)	0.208
< 50	227 (27.5%)	47 (32.2%)	0.251	133 (31.4)	30 (35.3)	0.488	25 (35.7)	3 (13.0)	0.040
≥50	597 (72.5%)	99 (67.8%)		290 (68.6)	55 (64.7)		45 (64.3)	20 (87.0)	
**Race**									
White	669 (81.2)	114 (78.1)	0.636	343 (81.1)	67 (78.8)	0.686	55 (78.6)	18 (78.3)	0.754
Black	62 (7.5)	16 (11.0)		38 (9.0)	11 (12.9)		6 (8.6)	1 (4.3)	
Asian/PI	84 (10.2)	15 (10.3)		37 (8.7)	7 (8.2)		8 (11.4)	4 (17.4)	
AI/AN	3 (0.4)	0 (0.0)		4 (0.9)	0 (0.0)		0 (0.0)	0 (0.0)	
Unknown	6 (0.7)	1 (0.7)		1 (0.2)	0 (0.0)		1 (1.4)	0 (0.0)	
**T Stage**									
pT1	542 (65.8)	70 (47.9)	0.000	250 (59.1)	48 (56.5)	0.653	32 (45.7)	7 (30.4)	0.198
pT2	282 (34.2)	76 (52.1)		173 (40.9)	37 (43.5)		38 (54.3)	16 (69.6)	
**Grade**									
1	244 (29.6)	42 (28.8)	0.004	88 (20.8)	12 (14.1)	0.354	3 (4.3)	0 (0.0)	0.438
2	508 (61.7)	83 (56.8)		239 (56.5)	50 (58.8)		32 (45.7)	8 (34.8)	
3	53 (6.4)	21 (14.4)		91 (21.5)	23 (27.1)		32 (45.7)	15 (65.2)	
Unknown	19 (2.3)	0 (0.0)		5 (1.2)	0 (0.0)		3 (4.3)	0 (0.0)	
**Histology**									
IDC	607 (73.7)	96 (65.8)	0.160	328 (77.5)	69 (81.2)	0.339	67 (95.7)	20 (87.0)	0.179
ILC	125 (15.2)	31 (21.2)		48 (11.3)	10 (11.8)		1 (1.4)	1 (4.3)	
IDC/ILC	78 (9.5)	15 (10.3)		42 (9.9)	4 (4.7)		1 (1.4)	2 (8.7)	
Other	14 (1.7)	4 (2.7)		5 (1.2)	2 (2.4)		1 (1.4)	0 (0.0)	
**Chemotherapy**									
No/Unknown	714 (86.7)	119 (81.5)	0.100	247 (58.4)	31 (36.5)	0.000	19 (27.1)	2 (8.7)	0.066
Yes	110 (13.3)	27 (18.5)		176 (41.6)	54 (63.5)		51 (72.9)	21 (91.3)	
**Year of Diagnosis**									
Before 2014	516 (62.6)	86 (58.9)	0.394	301 (71.2)	50 (58.8)	0.025	44 (62.9)	13 (56.5)	0.588
2014 and there after	308 (37.4)	60 (41.1)		122 (28.8)	35 (41.2)		26 (37.1)	10 (43.5)	

*TNM stage was according to the 8th edition of the American Joint Commission on Cancer (AJCC) staging system*.

*Asian/PI, Asian or Pacific Islander; AI/AN, American Indian or Alaskan Native; PMRT, Post-mastectomy radiotherapy*.

### Treatment Information

All patients in this study underwent mastectomy. As shown in Table 1, in the Intermediate-Risk group, more patients in the PMRT subgroup received chemotherapy (63.5 vs. 41.6%, *P* = 0.000), while in the High-Risk group, more patients in the PMRT subgroup received chemotherapy with a borderline significance (91.3 vs. 72.9%, *P* = 0.066). As all patients in this study had ER-positive and HER2-negative breast cancer, all should receive endocrine therapy as recommended while Trastuzumab was worthless for those patients. However, the SEER database does not contain information on endocrine or target therapy of breast cancer patients, and this is one of the limitations of our study.

### Clinical Outcomes

**Figure 2:**
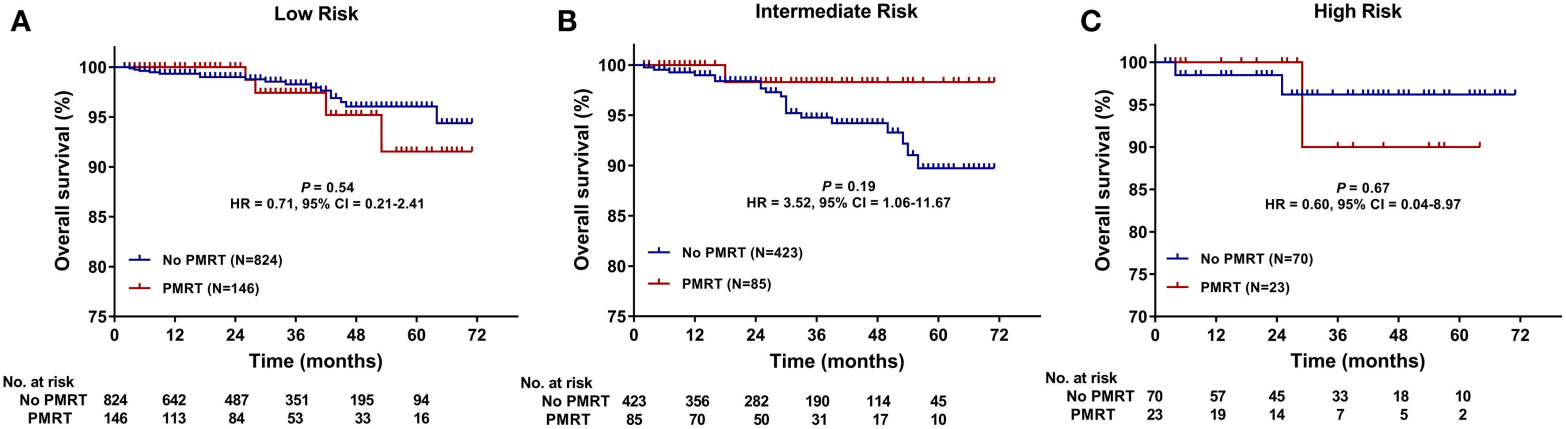
Overall survival (OS) of the PMRT subgroup and the No PMRT subgroup in the Low- **(A)**, Intermediate- **(B)**, and High-Risk **(C)** group. CI, Confidence interval; HR, hazard ratio; PMRT, Post-mastectomy radiotherapy.

The median follow-up time was 30 months (range, 2–71 months) for all patients. The 5-years OS for all patients was 93.8 ± 1.1%. There was no significant difference in follow-up time between the no PMRT subgroup and PMRT subgroup in the Low- (30 vs. 28 months, *P* = 0.323), Intermediate- (32 vs. 28 months, *P* = 0.235), or High-Risk group (31.5 vs. 26 months, *P* = 0.081). OS was comparable between the PMRT subgroup and no PMRT subgroup in all three groups (Figure 2). In the Low-Risk group, 5-years OS was 96.1% in the no PMRT group and 91.5% in the PMRT group (*P* = 0.539) (Figure 2A). In the Intermediate-Risk group, 5-years OS was 89.7% in the no PMRT group and 98.3% in the PMRT group (*P* = 0.189) (Figure 2B). And in the High-Risk group, 5-years OS was 96.2% in the no PMRT group and 90.0% in the PMRT group (*P* = 0.671) (Figure 2C). We also compared OS between the PMRT group and no PMRT group in all patients and did not see any benefit for PMRT in this group of patients as a whole (*P* = 0.757) (**Figure 4A**).

**Figure 3:**
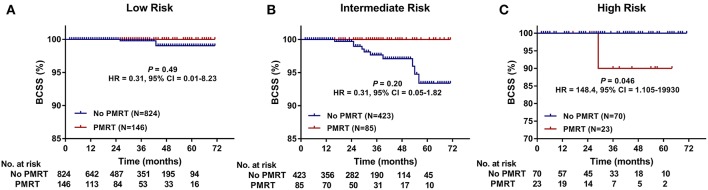
Breast cancer-specific survival (BCSS) of the PMRT subgroup and the No PMRT subgroup in the Low- **(A)**, Intermediate- **(B)**, and High-Risk **(C)** group. BCSS, Breast cancer-specific survival; CI, Confidence interval; HR, hazard ratio; PMRT, Post-mastectomy radiotherapy.

**Figure 4:**
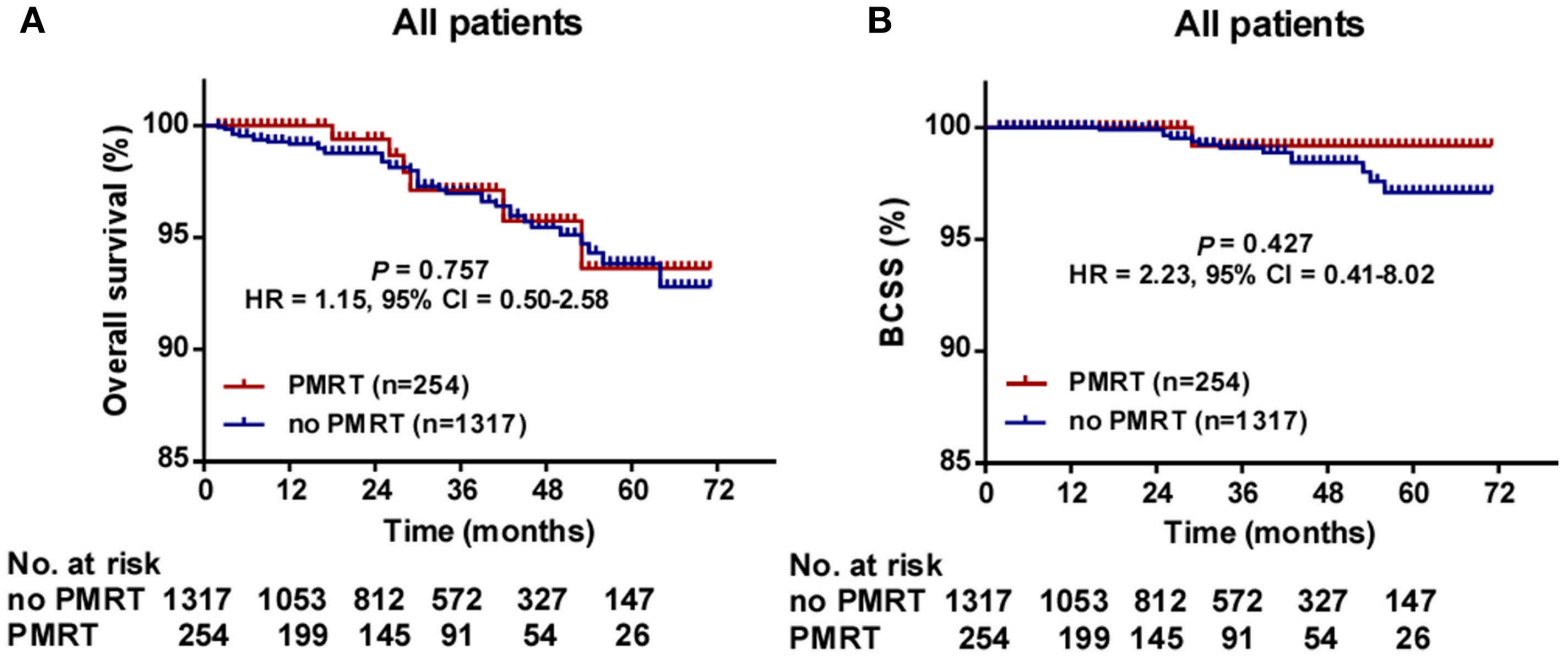
Overall survival (OS) **(A)** and Breast cancer-specific survival (BCSS) **(B)** of the PMRT subgroup and the No PMRT subgroup in all patients. BCSS, Breast cancer-specific survival; CI, Confidence interval; HR, hazard ratio; PMRT, Post-mastectomy radiotherapy.

Furthermore, we compared BCSS within each group. In the High-Risk group, BCSS of the no PMRT subgroup seemed to be superior to that of the PMRT subgroup (5-years BCSS: 100.0 vs. 90.0%, *P* = 0.046) (Figure 3C). However, no significant difference was observed between the no PMRT and PMRT subgroup in the Low-Risk (5-years BCSS: 99.0 vs. 100.0%, *P* = 0.485) (Figure 3A) and Intermediate-Risk group (5-years BCSS: 93.4 vs. 100.0%, *P* = 0.195) (Figure 3B). Then we compared BCSS between the PMRT group and no PMRT group in all patients and again did not find any benefit for PMRT in this group of patients as a whole (*P* = 0.427) (Figure 4B).

Potential survival predictors, including age, race, T stage, grade, histology, whether or not PMRT was administered, whether or not chemotherapy was delivered, year of diagnosis, were analyzed using univariate analysis followed by Cox regression model in each group and all patients (Table 2). In the Low-Risk group, T stage, age, and race were entered into Cox multivariate regression model as they showed a *P* < 0.15 in the univariate analysis. T stage (HR = 3.032, 95% CI, 1.252–7.339, *P* = 0.014) was found to be an independent predictor for (OS) (Table 2). However, in the Intermediate-Risk and High-Risk group, we did not find any variable to be an independent predictor for (OS) (Table 2). In addition, Cox regression analysis showed that PMRT was not an independent predictor for (OS) either (*P* = 0.643).

**Table 2:** Univariate and multivariate analysis of prognostic factors for overall survival in the Low-, Intermediate-, and High-Risk group.

**Variable**	**Low-Risk**			**Intermediate-Risk**			**High-Risk**		
	**Univariate analysis**	**Multivariate analysis**		**Univariate analysis**	**Multivariate analysis**		**Univariate analysis**	**Multivariate analysis**	
	***P*-value**	***P*-value**	**HR (95%CI)**	***P*-value**	***P*-value**	**HR (95%CI)**	***P*-value**	***P*-value**	**HR (95%CI)**
**Age** (< 50 vs. ≥50)	0.027	0.063	6.769 (0.900–50.915)	0.058	0.094	3.533 (0.806–15.486)	0.136		
**Race**	0.083	0.119		0.012	0.090		0.000	1.000	
Race (1)	0.041	0.958		0.118	0.958		0.481		
Race (2)	0.005	0.950		0.044	0.948		0.627		
Race (3)	0.830	0.954		0.597	0.958		0.560		
Race (4)	0.742	0.996		0.004	0.938		/		
**T stage** (pT1 vs. pT2)	0.005	0.014	3.032 (1.252–7.339)	0.545			0.445		
**Grade**	0.586			0.387			0.514		
**Histology**	0.841			0.303			0.022	1.000	
Histology (1)	0.749			0.064			0.102		
Histology (2)	0.754			0.347			0.750		
Histology (3)	0.513			0.180			0.002		
**PMRT**	0.539			0.190			0.671		
**Chemotherapy**	0.743			0.708			0.495		
**Year of Diagnosis** (before 2014 vs. after 2014)	0.946			0.632			0.182		

*TNM stage was according to the 8th edition of the American Joint Commission on Cancer (AJCC) staging system*.

*CI, Confidence interval; HR, hazard ratio; PMRT, Post-mastectomy radiotherapy*.

## Discussion

In this study of a large cohort of women with T1-2 N1mic ER-positive HER2-negative breast cancer who underwent mastectomy from the SEER database, the results showed that the 21-gene RS assay was not able to predict the benefit of PMRT for OS. Multivariate analysis showed PMRT was not an independent predictor for (OS) in the Low-, Intermediate-, or High-Risk group. This is an interesting and practical finding which suggests that decisions about PMRT should not solely be based on the 21-gene RS in this group of breast cancer patients.

It is still controversial whether PMRT is necessary for women with T1-2 breast cancer and one-to-three positive axillary lymph nodes. Data from clinical trials and meta-analyses have demonstrated that PMRT reduces (LRR) and improves (OS) in patients with 1-3 positive nodes, even when systemic therapy was given (2–6). However, it has not been universally accepted that if every patient with T1-2 and 1-3 positive nodes should receive PMRT. A joint statement by ASCO, ASTRO, and SSO proposes that some subsets of these patients are likely to have very low risk of LRR and the absolute benefit of PMRT might be outweighed by its potential toxicities (7). For patients with lymph node micrometastases, this concept may be even more relevant since their prognosis might be between those with N0 disease and those with lymph node macrometastases (8, 10, 12–14). However, there is a paucity of studies focusing on the effect of PMRT on this group of breast cancer patients. At the same time, the 21-gene RS has been validated in T1-2 N1 breast cancer to be prognostic of LRR, DFS, and OS (17–21), and a recent published study shows patients with different RS might derive different benefit from PMRT in T1-2 N1 ER-positive breast cancer patients (22). For the reasons above, we did this study to find out if the 21-gene RS assay was a potential indicator for PMRT decision-making in this group of breast cancer patients. To our knowledge, this is the first report evaluating the value of 21-gene RS on decision-making of PMRT for women with T1-2 N1mic ER-positive HER2-negative breast cancer who underwent mastectomy in a population-based cohort.

As this was a retrospective study, some of the baseline clinical characteristics of the PMRT and no PMRT subgroup in the Low-, Intermediate-, and High-Risk groups were unbalanced. For example, in the Low-Risk group, more patients in the PMRT subgroup had T2 and Grade 3 disease, while in the Intermediate-, and High-Risk groups more patients in the PMRT subgroup received chemotherapy. And in fact, more patients in the High-Risk groups received PMRT than in the Low- and Intermediate-Risk group. Possible explanations are as follows. First, as this was a retrospective study, selection bias was inevitable, especially when the cohort size was not very large. The second possible reason, which was very important, was that physicians were more likely to choose more aggressive treatment strategies, such as PMRT and adjuvant chemotherapy, for patients with a higher 21-gene RS or larger tumor or higher pathologic grade as they believed those patients were at a higher risk of disease progression, recurrence, or metastasis. However, until now, there was no sufficient evidence supporting the use of PMRT in T1-2 N1mic M0 ER-positive and HER2-negative breast cancer patients according to 21-gene RS.

The axillary lymph node status has been known to be one of the most important prognostic factors in patients with breast cancer. Some studies indicate that even the presence of micrometastasis in axillary lymph nodes can affect survival (8–12), while others show micrometastasis disease does not adversely affect patients' outcome (13–16, 23). Previous studies reported that the 5 year,-OS of patients with axillary lymph node micrometastasis ranged from 84.7 to 97% (24–27). In line with those results, the 5-years OS of all patients in the present study was 93.8%.

Data of our study showed no significant differences in OS between patients in the PMRT and no PMRT subgroup in the Low-, Intermediate-, and High-Risk group. BCSS were also comparable between the PMRT and no PMRT subgroup in the Low- and Intermediate-Risk group. In the High-Risk group, BCSS of the no PMRT subgroup seemed to be more favorable than that of the PMRT subgroup. However, we should be cautious with that disparity. There are several possible explanations. First, as the number of patients in the High-risk group was so limited, it might be caused by selection bias. Second, as this is a retrospective study without a unified protocol, probably treating physicians would suggest patients with more risk factors to undergo PMRT more frequently. As a result, patients in the PMRT subgroup would have worse prognosis. A third reason is that as patients with a higher recurrence score have a higher risk of distant failure, they might not derive much benefit from PMRT. And on the other hand, PMRT could also bring some toxicities, such as radiation pneumonitis and radiation-induced heart disease. All together, the moderate toxicities caused by PMRT might outweight its limited benefit. What's more, further Cox multivariate regression analysis showed no prognostic value of PMRT for OS in any of the three groups. Recently, Goodman et al. reported the result of their study on the value of 21-gene RS assay on predicting benefit of PMRT in T1-2N1 breast cancer patients (22). The result showed that patients with a low RS derived a greater survival benefit from PMRT than those with an intermediate or high RS in T1-2N1 ER-positive breast cancer patients. They supposed that it might be due to a low competing risk of subclinical micrometastatic disease in patients with a low RS at diagnosis resulting in improved translation of locoregional control to a survival benefit. On the other hand, patients with an intermediate or high RS who are at a higher risk for subclinical micrometastatic disease may not derive a survival benefit from a locoregional treatment due to a competing risk of distant failure. However, in the present study we did not see any survival benefit of PMRT for patients with T1-2 N1mic ER-positive HER2-negative breast cancer in any of the three groups with different 21-gene RS, which seemed to be inconsistent with the hypothesis proposed by Goodman et al. Several factors might account for this discrepancy. First, in the present study, we only enrolled patients with axillary lymph node micrometastasis and ER-positive HER2-negative disease. Such patients are usually at a very low risk of distant metastasis as well as (LRR) and they might not derive a survival benefit from a locoregional treatment due to a very low risk of locoregional failure. As a result, those patients will probably not derive benefit from adjuvant radiotherapy. Second, radiation-related toxicities, such as radiation pneumonitis, might counterweigh the benefit of PMRT for locoregional control. Third, as this was a retrospective cohort study, selection bias existed, which has been mentioned above, and might interfere with the result.

There are several limitations in our study, including the retrospective nature, the inadequate follow-up period, and relative small sample size especially within the High-risk group. In addition, the SEER database does not provide information on several quite important variables, such as reception of endocrine therapy, the exact chemotherapy regimen and its sequence with surgery, as well as the radiation area and radiation dose. What's more, we could not assess the consideration of physicians and the negotiation between physicians and patients on treatment decision-making. And at last, we could not get information on (LRR) and distant metastasis of the patients from the SEER database, which prevented us to explore the exact role of PMRT in this study.

## Conclusion

In conclusion, this study showed that the 21-gene RS assay was not able to predict the benefit of PMRT for OS in women with T1-2 N1mic ER-positive HER2-negative breast cancer. Therefore, decisions regarding PMRT should not be based solely on the 21-gene RS in this group of breast cancer patients. However, further prospective larger sample-size trials are warranted.

## Author Contributions

H-XL and S-GW guarantee for the integrity of the manuscript and contributed to the concept and design. W-WZ and QT contributed to analysis and interpretation of the data and the drafting and revision of the manuscript. Z-YH, F-YL, and YD contributed to the concept and design, and editing and revision of the manuscript. J-YS, XH, Z-QL, and J-PD contributed to data collection, and editing, and revision of the manuscript. All authors read and approved the final manuscript.

### Conflict of Interest Statement

The authors declare that the research was conducted in the absence of any commercial or financial relationships that could be construed as a potential conflict of interest.

## Footnotes

^1^https://seer.cancer.gov/statfacts/html/breast.html
